# Applicability of modified weibull extension distribution in modeling censored medical datasets: a bayesian perspective

**DOI:** 10.1038/s41598-022-21326-w

**Published:** 2022-10-13

**Authors:** Navid Feroze, Uroosa Tahir, Muhammad Noor-ul-Amin, Kottakkaran Sooppy Nisar, Mohammed S. Alqahtani, Mohamed Abbas, Rashid Ali, Anuwat Jirawattanapanit

**Affiliations:** 1 Department of Statistics, The University of Azad Jammu and Kashmir, Muzaffarabad, Pakistan; 2 Department of Statistics, COMSATS University Islamabad-Lahore Campus, Lahore, Pakistan; 3 Department of Mathematics, College of Arts and Sciences, Prince Sattam Bin Abdulaziz University, Wadi Aldawaser, Saudi Arabia; 4 Radiological Sciences Department, College of Applied Medical Sciences, King Khalid University, Abha, 61421 Saudi Arabia; 5 BioImaging Unit, Space Research Centre, Michael Atiyah Building, University of Leicester, Leicester, LE1 7RH UK; 6 Electrical Engineering Department, College of Engineering, King Khalid University, Abha, 61421 Saudi Arabia; 7 Electronics and communications Department, College of Engineering, Delta University for Science and Technology, Gamasa, 35712 Egypt; 8 School of Mathematics and Statistics, HNP LAMA, Central South University, Changsha, 410083 Hunan People’s Republic of China; 9 Department of Mathematics, Faculty of Science, Phuket Rajabhat University (PKRU), 6 Thepkasattree Road, Raddasa, Phuket, 83000 Thailand

**Keywords:** Cancer, Computational biology and bioinformatics, Diseases, Health care, Medical research, Mathematics and computing

## Abstract

There are some contributions analyzing the censored medical datasets using modifications of the conventional lifetime distribution; however most of the said contributions did not considered the modification of the Weibull distribution (WD). The WD is an important lifetime model. Due to its prime importance in modeling life data, many researchers have proposed different modifications of WD. One of the most recent modifications of WD is Modified Weibull Extension distribution (MWED). However, the ability of MWED to model the censored medical data has not yet been explored in the literature. We have explored the suitability of the model in modeling censored medical datasets. The analysis has been carried out using Bayesian methods under different loss functions and informative priors. The approximate Bayes estimates have been computed using Lindley’s approximation. Based on detailed simulation study and real life analysis, it has been concluded that Bayesian methods performed better as compared to maximum likelihood estimates. In case of small samples, the performance of Bayes estimates under ELF and informative prior was the best. However, in case of large samples, the choice of prior and loss function did not affect the efficiency of the results to a large extend. The MWED performed efficiently in modeling real censored datasets relating to survival times of the leukemia and bile duct cancer patients. The MWED was explored to be a very promising candidate model for modeling censored medical datasets.

## Introduction

The literature contains many valuable contributions for analysis of lifetime data using different modifications of the Weibull distribution. Silva et al.^1^ introduced beta modified Weibull distribution and showed that it is suitable in modeling data with monotone failure rates. Almalki and Yuan^2^ introduced a new modified Weibull distribution and estimated its model parameters based on order statistics using moment estimates, MLE and Bayes estimates. Sarhan and Apalo^3^ proposed exponentiated modified Weibull extension distribution and discussed its applications in different fields. Peng and Yan^4^ introduced extended Weibull distribution, estimated the model parameters and explored applicability of the model. Ahmad and Iqbal^5^ developed the generalized flexible Weibull extension distribution and compared its modeling capabilities with some conventional life models. El-Morshedy et al.^6^ proposed three parametric exponentiated inverse flexible Weibull extension distribution. The proposed model was shown to be better than other modifications of Weibull distributions in modeling life datasets. Tahir et al.^7^ introduced transmuted Weibull-Pareto and evaluated its important properties. Lindley-Weibull distribution was introduced by Cordeiro et al.^8^, as a better alternate to Weibull distribution.

The Bayes estimation for Weibull distributions and its modified forms has attracted many researchers recently. Feroze et al.^9^ used LA, Tierney and Kadane’s approximation, Gibbs sampler and importance sampling for Bayesian analysis of right censored Weibull distribution. Kaur et al.^10^ considered LA for Bayesian estimation of generalized inverse Weibull distribution. Nofal et al.^11^ introduced transmuted exponentiated additive Weibull distribution and claimed it as more flexible model to analyze the real data under classical and Bayesian methods. Yari and Tondpour^12^ introduced burr XII exponential distribution. The MLE and Bayesian methods were used to estimate the model parameters. The Bayes estimates were obtained using MCMC and LA methods. Saboor et al.^13^ discussed the estimation for parameters of modified beta modified Weibull distribution using MLE and Bayes methods. Shahzad et al.^14^ considered the estimation of beta exponentiated modified Weibull distribution using MLE. Xu and Gui^15^ introduced entropy estimation of inverse Weibull distribution by using type-II progressive hybrid censoring via LA. Rao and Mbwambo^16^ used MCMC method to analyze different properties of exponentiated inverse Rayleigh distribution. It was shown that MCMC method performs better than MLE. Babacan and Kaya^17^ used LA and method of MCMC for estimation of different properties of Weibull distribution.

The researchers have frequently considered various modifications of lifetime models for modeling censored medical datasets, however majority of the proposed models were not the generalizations of the Weibull distribution. The modified Weibull extension model (MWED), having bathtub hazard rate, has been recently introduced by Xie et al*.*^18^. The additional feature of MWED is that the confidence interval for the shape parameter and joint confidence interval can be derived explicitly. Xie et al*.*^18^ proved the superiority of MWED over Weibull and exponentiated Weibull model, in modeling lifetime data. The applications of the MWED in satellite reliability engineering have been explored by Yang et al.^19^.

From the above discussion, it can be assessed that classical and Bayesian estimation for the modifications of Weibull distribution is receiving significant interest of the researchers. Exploring new models for modeling censored medical datasets is quite important. Different modifications of the Weibull distribution have been employed to model the medical datasets. The few recent studies include the followings. Klakattawi^20^ dealt with survival analysis of cancer patients using extended Weibull model. Wahed et al.^21^ proposed a generalization of the Weibull distribution to model the survival times of the breast cancer patients. Alahmadi et al.^22^ considered weighted Weibull distribution to model COVID-19 data. Adam et al.^23^ introduced modified Weibull distribution for biomedical signal denoising. However, the analysis of MWED using censored medical datasets has not been discussed in the literature yet. In addition, the Bayesian analysis of medical datasets using MWED is still lacking in literature. This paper bridges these gaps by proposing Bayesian analysis of censored medical datasets using MWED. The informative priors have been assumed for posterior estimation. In addition, different loss functions such as, squared error loss function (SELF), quadratic loss function (QLF), precautionary loss function (PLF) and entropy loss function (ELF) have been be used for the posterior estimation. The SELF is symmetric loss function while, QLF, PLF and ELF are asymmetric loss functions. The symmetric loss function is used in the situations where over-estimation and under-estimation are equally important. On the other hand, when either over-estimation or under-estimation is more important, an asymmetric loss function is used. As the posterior estimates do not have explicit forms, Lindley’s approximation (LA) has been used for the numerical solutions. LA is used to approximate the ratio of multiple integrals, when the analytical solutions are nor possible. The numerical computations have been done using Mathematica and R softwares. The suitability of the MWED in modeling real censored datasets regarding survival times of the leukemia and bile duct cancer patients has been explored.

## Materials and methods

The posterior estimation for the parameters of MWED has been considered informative priors. For posterior estimation of the parameters of the MWED, different symmetric and asymmetric loss functions such as, SELF, PLF, QLF and ELF, have been used. In order to obtain the numerical solutions, the LA method has been used. The performance of the proposed Bayes estimators has been compared under a simulation study. The applications of the MWED have been explored in medical field involving analysis of two censored real life datasets.

### Modified Weibull extension distribution (MWED)

This section includes the introduction of MWED. The MWED is very useful lifetime model, especially when the hazard rate has bathtub shape (Yang et al.^19^). In addition, the modeling of failure times and reliability using MWED is quite convenient due to its closed form expressions for cumulative distribution function (Xie et al.^18^). The additional feature of MWED is that the confidence interval for the shape parameter and joint confidence interval can be derived explicitly. Due to these features, the MWED is very suitable candidate to model the censored lifetimes. The analysis of applicability of MWED to model censored datasets relating to medical field can be very interesting. After defining the basic formulation about MWED in this Section, the estimates based MWED has been used to model the right censored medical datasets. The density function and some important characteristics of MWED have been reported in the following equations.

The probability density function (PDF) of the MWED is

1 $$f\left( {{\text{x|}} \theta ,\sigma ,\mu } \right) = { }\sigma \mu \left( {(x/\theta )^{\sigma - 1} } \right)Exp\left[ {(x/\theta )^{\sigma } + \mu \theta \left\{ {1 - { }Exp(x/\theta )^{\sigma } } \right\}} \right]$$

where $\theta ,\sigma ,\mu \ge 0$ are the parameters of the model and $x \ge$ 0.

The cumulative distribution function (CDF) of the MWED is

2 $${\text{F}}\left( {{\text{x|}} \theta ,\sigma ,\mu } \right) = { }1 - Exp\left[ {\mu \theta \left\{ {1 - Exp(x/\theta )^{\sigma } } \right\}} \right],\quad \theta ,\sigma ,\mu \ge 0,t \ge 0$$

The reliability function for the MWED is

3 $${\text{R}}\left( {{\text{x|}} \theta ,\sigma ,\mu } \right) = { }Exp \left[ {\mu \theta \left\{ {1 - Exp\left( {x/\theta } \right)^{\sigma } } \right\}} \right],\quad \theta ,\sigma ,\mu \ge 0,t \ge 0$$

The failure rate function for the MWED is

4 $$r\left( {{\text{x|}} \theta ,\sigma ,\mu } \right) = { }\sigma \left[ {\left( {x/\theta } \right)^{\sigma - 1} Exp\left( {x/\theta } \right)^{\sigma } } \right]$$

The quantile function of MWED is

5 $${\text{q}}\left( {{\text{x|}} \theta ,\sigma ,\mu } \right) = \theta \log \left[ {\left( {1 - \frac{1}{\sigma \theta }} \right) + \left( {1 - u} \right)} \right]^{1/\mu }$$

where θ is a scale parameter and σ, $\mu$ are shape parameters and ‘u’ is uniformly distributed over range (0, 1). This model has Weibull distribution as a special and asymptotic case, so it can be considered as a Weibull extension distribution. When σ ≥ 1 the hazard rate function is an increasing function and when σ ≤ 1 the hazard rate function is a bathtub-shaped function.

### Bayesian estimation of the MWED using right censored datasets

The important part of the Bayesian estimation is to obtain the likelihood function for the sampling distribution. The likelihood function under type-II censored samples can be defined as. Suppose that ‘n’ items are put on a test and the test was terminated when the ‘r’ failures were observed. Hence the ‘n − r’ items were type-II right censored. Then the likelihood function for the said type-II right censored dataset is

6 $$L\left( {{\varvec{x}}{|}\theta ,\sigma , \mu } \right) = \mathop \prod \limits_{i = 1}^{r} f\left( {x_{i} } \right)\left[ {1 - F\left( {x_{r} } \right)} \right]^{n - r}$$

The Likelihood function under censored samples

7 $$\begin{aligned} & L\left( {\theta ,\sigma , \mu ;\user2{ x }} \right) = \sigma^{r} \mu^{r} \mathop \prod \limits_{i = 1}^{r} \left( {\frac{{x_{i} }}{\theta }} \right)^{\sigma - 1} Exp\left[ {\left( {\frac{{x_{i} }}{\theta }} \right)^{\sigma } + \mu \theta \left\{ {1 - Exp\left( {\frac{{x_{i} }}{\theta }} \right)^{\sigma } } \right\}} \right] \\ & \quad \times Exp\left[ {\mu \theta \left( {n - r} \right)\left\{ {1 - Exp\left( {\frac{{x_{r} }}{\theta }} \right)} \right\}} \right] \\ \end{aligned}$$

#### Prior and posterior distributions

The additional advantage of the Bayesian methods is that they can incorporate the prior information to update the current state of knowledge about the model parameters. This study will include the assumption of non-informative and informative priors for the derivation of Bayes estimates under different loss functions.

The joint informative prior assuming gamma prior for each parameter of MWED is.

8 $${\text{g}}\left({\varvec{\varPsi}}\right){ } \propto { }\theta^{{a_{{1{ }}} - 1}} { }\sigma^{{a_{{2{ }}} - 1}} { }\mu^{{a_{{3{ }}} - 1}} { }e^{{ - \left( {\theta b_{1} + \sigma b_{2} + \mu b_{3} } \right)}}$$

where $a_{{1{ }}} ,$ $a_{{2{ }}} ,a_{{3{ }}} ,b_{1} , b_{2} ,b_{3}$ are hyper-parameters.

The values of the hyper-parameters have been chosen by using prior mean approach. In prior mean, the values of the prior-parameters are selected in the way that prior means becomes approximately equal to the true parametric values. In case of real datasets, the true parametric values are not available, so the values of the hyper-parameters has been chosen to be so that the prior mean become approximately equal to the MLEs for the model parameters. The MLE estimators have been obtained by maximizing (7) with respect to model parameters. The R Code for obtaining MLEs has been given in Supplementary information.

The posterior distribution under Gamma prior

9 $$\begin{aligned} & P\left( {\theta ,\sigma , \mu {|}{\varvec{x}}} \right) \propto \sigma^{r} \mu^{r} \mathop \prod \limits_{i = 1}^{r} \left( {\frac{{x_{i} }}{\theta }} \right)^{\sigma - 1} Exp\left[ {\left( {\frac{{x_{i} }}{\theta }} \right)^{\sigma } + \mu \theta \left\{ {1 - Exp\left( {\frac{{x_{i} }}{\theta }} \right)^{\sigma } } \right\}} \right] \\ & \quad \times {\kern 1pt} \left[ {\mu \theta \left( {n - r} \right)\left\{ {1 - Exp\left( {\frac{{x_{r} }}{\theta }} \right)} \right\}\left( {{\uptheta }^{{a_{{1{ }}} - 1}} { }\sigma^{{a_{{2{ }}} - 1}} { }\mu^{{a_{{3{ }}} - 1}} { }e^{{ - \left( {\theta b_{1} + \sigma b_{2} + \mu b_{3} } \right)}} { }} \right)} \right] \\ \end{aligned}$$

As the closed form expressions for the Bayes estimates of model parameters under SELF, PLF, QLF and ELF are not possible, the Bayesian approximate method, namely, Lindley’s approximate has been used to obtain the numerical solutions for model parameters under the said loss functions. The results under Bayes estimates have also been compared with most commonly used classical method, namely, MLE.

#### Loss functions

The introduction of the loss functions used in the study is presented in the following. The SELF is defined as: ${\text{L}}\left( {\theta ,{ }\theta_{{{\text{SELF}}}} } \right) = \left( {\theta - \theta_{{{\text{SELF}}}} } \right)^{2}$. The Bayes estimator and posterior risks under SELF are $\theta_{{{\text{SELF}}}} = {\text{E}}\left( \theta \right){ }$ and ${\text{P}}\left( {\theta_{{{\text{SELF}}}} } \right) = {\text{E}}\left( {\theta^{2} } \right) - \left\{ {{\text{E}}\left( \theta \right)} \right\}^{2}$, respectively. The PLF is defined as:$\user2{ }{\text{L}}\left( {\theta_{{{\text{PLF}}}} ,{ }\theta } \right) = { }\frac{{{ }\left( {\theta_{{{\text{PLF}}}} - \theta } \right)^{2} }}{{\theta_{{{\text{PLF}}}} }} { }$. The Bayes estimator and posterior risk considering PLF are $\theta_{{{\text{PLF}}}} = \left\{ {{\text{E}}\left( {\theta^{2} } \right)^{1/2} } \right\}{ }$ and ${\text{P}}\left( {\theta_{{{\text{PLF}}}} } \right) = 2\left\{ {\theta_{{{\text{PLF}}}} - {\text{E}}\left( \theta \right)} \right\}$, respectively. The QLF is defined as:${\text{L}}\left( {\theta ,{ }\theta_{{{\text{QLF}}}} } \right) = { }\left[ {\frac{{\theta - \theta_{{{\text{QLF}}}} }}{\theta }} \right]^{2}$. The Bayes estimator and posterior risk using QLF are $\theta_{{{\text{QLF}}}} = {\text{E}}\left( {\theta^{ - 1} } \right)\left\{ {{\text{E}}\left( {\theta^{ - 2} } \right)} \right\}^{ - 1}$ and ${\text{P}}\left( {\theta_{{{\text{QLF}}}} } \right) = {\text{E}}\left[ {\frac{{\theta - \theta_{{{\text{QLF}}}} }}{\theta }} \right]^{2} = 1 - \left[ {\frac{{\left\{ {{\text{E}}\left( {\theta^{ - 1} } \right)} \right\}^{2} }}{{{\text{E}}\left( {\theta^{ - 2} } \right)}}} \right]$, respectively. The ELF is defined as:${\text{L}}\left( {\theta ,{ }\theta_{{{\text{ELF}}}} } \right) = \left( {\frac{{\theta_{{{\text{ELF}}}} }}{\theta }} \right) - \ln \left( {\frac{{\theta_{{{\text{ELF}}}} }}{\theta }} \right) - 1$. The Bayes estimator and posterior risk for ELF are $\theta_{{{\text{ELF}}}} = \left[ {{\text{E}}\left( {\theta^{ - 1} } \right)} \right]^{ - 1}$ and ${\text{P}}\left( {\theta_{{{\text{ELF}}}} } \right) = {\text{E}}\left\{ {{\text{L}}\left( {\theta_{{{\text{ELF}}}} ,{ }\gamma } \right)} \right\} = {\text{E}}\left\{ {\ln \left( \theta \right)} \right\} - {\text{ln}}\left( {\theta_{{{\text{ELF}}}} } \right)$, respectively.

#### Lindley’s approximation (LA)

Having sufficiently large samples, Lindley^24^ proposed that function of the form

10 $$I\left( \Psi \right) = E\left[ {h\left( \Psi \right)} \right] = \frac{{\mathop \smallint \nolimits_{\phi }^{\infty } h\left( \Psi \right)e^{{I\left( {\Psi {|}x} \right) + G\left( \Psi \right)}} d\Psi }}{{\mathop \smallint \nolimits_{\phi }^{\infty } e^{{I\left( {\Psi {|}x} \right) + G\left( \Psi \right)}} d\Psi }}$$

where $\Psi = \left( {\theta , \sigma ,\mu } \right)$, $h\left( \Psi \right)$ is some function involving $\Psi$, $I\left( {\Psi {|}x} \right)$ is the logarithmic of likelihood function and $G\left( \Psi \right)$ in the logarithmic of $g\left( \Psi \right)$ given in (8), can be given in the following form

11 $$I\left( \Psi \right) = h\left( {\hat{\Psi }} \right) + \left( {h_{1 } v_{1} + h_{2 } v_{2} + h_{3 } v_{3} + v_{4 } + v_{5 } } \right) + \frac{1}{2}\left( {K_{1} W_{1} + K_{2 } W_{2} + K_{3 } W_{3} } \right)$$

where $\widehat{\Psi }$ is MLE of the parametric set $\Psi$, $W_{t} = h_{1 } S_{t1} + h_{2 } S_{t2} + h_{3 } S_{t3}$,

$$\begin{aligned} & K_{t } = S_{11} L_{11t} + S_{22} L_{22t} + S_{33} L_{33t} + 2S_{12} L_{12t} + 2S_{13} L_{13t} + 2S_{23} L_{23t } , \\ & v_{t} = A_{1} S_{t1} + A_{2} S_{t2} + A_{3} S_{t3 } , t = 1, 2, 3, \\ & v_{6} = h_{12 } S_{12} + h_{13 } S_{13} + h_{23 } S_{23} + h_{23 } S_{23} , \\ & v_{7} = \frac{1}{2}\left( {h_{11 } S_{11} + h_{22 } S_{22} + h_{33 } S_{33} } \right), \\ & A_{t} = \frac{\partial G\left( \Psi \right)}{{\partial \Psi_{t} }}, \;\;t = 1, 2, 3, \Psi = \left( {\theta , \sigma ,\mu } \right), \\ & h_{tu} = \frac{{\partial^{2} h\left( \Psi \right)}}{{\partial \Psi_{t} \partial \Psi_{u} }},\;\;L_{tu} = \frac{{\partial^{2} L\left( \Psi \right)}}{{\partial \Psi_{t} \partial \Psi_{u} }},\;\;t, u = 1, 2, 3, \;\;L_{tuk} = \frac{{\partial^{3} l\left( \Psi \right)}}{{\partial \Psi_{t} \partial \Psi_{\mu } \partial \Psi_{k} }},\;\;t,u,k = 1, 2, 3, \\ \end{aligned}$$

and ${S}_{tu}$ is the ${\left(t , u\right)}^{th}$ element of the inverse of the matrix $\left\{{L}_{tu}\right\}$, where the elements of $\left\{{L}_{tu}\right\}$ will be evaluated using MLEs of the model parameters.

## Results

This section deals with the analytical and numerical estimation for the parameters of MWED using MLE and Bayesian method. The Bayes estimates have been obtained using different loss functions and informative priors. The LA has been used to obtain the numerical results for the Bayes estimates. The performance of different estimates has been compared using different simulated datasets. The suitability of the MWED has been explored in modeling the censored medical datasets. In particular, two censored medical datasets have been used for analysis.

### Simulation study using right censored datasets

The MLEs, Bayes estimates (BEs) and amounts of mean square errors (MSEs) for MWED under different loss functions SELF, PLF, QLF and ELF using different parametric spaces and sample sizes have been reported in this section. The results using censored simulated datasets have been reported in Tables 1, 2, 3, 4, 5, 6, 7, 8, 9, 10, 11 and 12. The simulated datasets have been generated using different sample sizes and different true parametric values. In particular, the samples of size 20, 50 and 100 have been generated from the MWED for analysis. The samples have been assumed to be 20% right censored in all samples. The inverse transformation has been employed to generate the said samples. The numerical values for the prior parameters have been chosen using prior mean methodology. The prior mean approach chooses the values of the hyper-parameters in such a way that prior mean approximate to true parametric value. The comparison between MLE and Bayesian estimation methods has been carried out using the amount of MSEs associated with respective estimates. In Tables, the amount of MSEs has been presented in the bold fonts.

**Table 1:** MLEs, BEs and MSEs for MWED using θ = 1, σ = 1 and μ = 1.

n		MLE	SELF	PLF	QLF	ELF
20	θ	1.1048	1.211	1.0944	1.1072	1.1488
		**0.5177**	**0.1597**	**0.1038**	**0.145**	**0.1386**
	σ	1.1291	1.1186	1.1054	1.1474	1.1187
		**0.6739**	**0.0698**	**0.0633**	**0.2049**	**0.0406**
	μ	1.1813	1.1377	1.1427	1.1527	1.0760
		**1.1853**	**0.1073**	**0.1146**	**0.0593**	**0.0342**
50	θ	1.0919	1.1622	1.0836	1.0679	1.0869
		**0.3290**	**0.1084**	**0.0897**	**0.0775**	**0.0886**
	σ	1.1217	1.0836	1.0986	1.1015	1.0868
		**0.2847**	**0.0483**	**0.0541**	**0.1345**	**0.0236**
	μ	1.1678	1.1030	1.1116	1.1386	1.0576
		**1.0115**	**0.0344**	**0.0469**	**0.0377**	**0.0178**
100	θ	1.0742	1.1076	1.0720	1.0449	1.0283
		**0.1430**	**0.0170**	**0.0119**	**0.0344**	**0.0118**
	σ	1.0923	1.0388	1.0291	1.0125	1.0393
		**0.1559**	**0.0026**	**0.0018**	**0.0071**	**0.0079**
	μ	1.1218	1.0453	1.0784	1.0757	1.0256
		**0.5557**	**0.0113**	**0.0124**	**0.0103**	**0.0066**

**Table 2:** MLEs, BEs and MSEs for MWED using θ = 1, σ = 2 and μ = 1.

n		MLE	SELF	PLF	QLF	ELF
20	θ	1.0939	1.0722	1.0856	1.0964	1.0781
		**0.8806**	**0.0339**	**0.0342**	**0.0352**	**0.0187**
	σ	2.1138	2.1026	2.0929	2.1249	2.1242
		**0.4525**	**0.5188**	**0.2436**	**0.0878**	**0.0539**
	μ	1.1350	1.0736	1.1137	1.0783	1.0729
		**1.0225**	**0.0934**	**0.0933**	**0.0822**	**0.0425**
50	θ	1.0878	1.0544	1.0504	1.0884	1.0715
		**0.5027**	**0.0059**	**0.0137**	**0.0182**	**0.0062**
	σ	2.1041	2.0444	2.0837	2.0631	2.1147
		**0.3627**	**0.0371**	**0.0885**	**0.0349**	**0.0387**
	μ	1.0941	1.0689	1.0945	1.063	1.0528
		**0.7252**	**0.0263**	**0.0404**	**0.0381**	**0.0254**
100	θ	1.0348	1.0037	1.0041	1.0499	1.0476
		**0.2317**	**0.0008**	**0.0010**	**0.0113**	**0.0026**
	σ	2.0648	2.0269	2.0280	2.0238	2.0518
		**0.3119**	**0.0061**	**0.0072**	**0.1388**	**0.0144**
	μ	1.0710	1.0243	1.0293	1.0458	1.0342
		**0.2822**	**0.0073**	**0.0079**	**0.0252**	**0.0075**

**Table 3:** MLEs, BEs and MSEs for MWED using θ = 1, σ = 2 and μ = 2.

n		MLE	SELF	PLF	QLF	ELF
20	θ	1.0927	1.0722	1.0743	1.1374	1.1252
		**1.0682**	**0.0142**	**0.0148**	**0.7354**	**0.0242**
	σ	2.0801	2.1122	2.1143	2.1127	2.0779
		**0.9407**	**0.2494**	**0.1373**	**0.2144**	**0.0276**
	μ	2.2013	2.1231	2.1296	2.1411	2.0979
		**1.4127**	**0.3088**	**0.1714**	**0.4371**	**0.0433**
50	θ	1.0790	1.0257	1.0277	1.0763	1.0337
		**0.7869**	**0.0055**	**0.0056**	**0.5436**	**0.0038**
	σ	2.0735	2.0546	2.0894	2.1081	1.9949
		**0.6464**	**0.0781**	**0.0449**	**0.1502**	**0.0107**
	μ	2.1722	2.0655	2.1058	2.1335	1.9888
		**1.1692**	**0.1263**	**0.0706**	**0.4039**	**0.0149**
100	θ	1.0601	1.0056	1.0007	1.0249	1.0003
		**0.2150**	**0.0028**	**0.0028**	**0.1827**	**0.0010**
	σ	2.0249	2.0112	2.0205	2.1012	1.9855
		**0.3468**	**0.0376**	**0.0190**	**0.0150**	**0.0051**
	μ	2.1208	2.0222	2.0374	2.0878	1.9807
		**0.6539**	**0.0622**	**0.0318**	**0.2820**	**0.0073**

**Table 4:** MLEs, BEs and MSEs for MWED using θ = 2, σ = 1, and μ = 1.

n		MLE	SELF	PLF	QLF	ELF
20	θ	2.0961	2.1491	2.103	2.1287	2.0943
		**1.2391**	**0.1435**	**0.0692**	**0.0564**	**0.0399**
	σ	1.1165	1.0904	1.1035	1.0703	1.0956
		**0.4704**	**0.0384**	**0.0409**	**0.0415**	**0.0261**
	μ	1.2117	1.0547	1.0574	1.1050	1.0873
		**1.0286**	**0.0331**	**0.0356**	**0.0970**	**0.0301**
50	θ	2.0920	2.0882	2.0633	2.1262	2.0797
		**0.7882**	**0.0841**	**0.0357**	**0.0360**	**0.0154**
	σ	1.1036	1.0714	1.0764	1.0423	1.0701
		**0.2211**	**0.0156**	**0.0157**	**0.0129**	**0.0111**
	μ	1.1893	1.0337	1.0257	1.0422	1.0501
		**0.7136**	**0.0187**	**0.0195**	**0.0122**	**0.0113**
100	θ	2.0709	2.0248	2.0181	2.0798	2.0501
		**0.1741**	**0.0434**	**0.0216**	**0.0150**	**0.0067**
	σ	1.0825	1.0186	1.0234	1.0135	1.0203
		**0.1734**	**0.0053**	**0.0054**	**0.0072**	**0.0039**
	μ	1.1446	1.0014	0.9909	1.0043	1.0016
		**0.3897**	**0.0076**	**0.0077**	**0.007**	**0.0032**

**Table 5:** MLEs, BEs and MSEs for MWED using θ = 2, σ = 2 and μ = 2.

n		MLE	SELF	PLF	QLF	ELF
20	θ	2.0851	2.1256	2.1132	2.1079	2.1443
		**2.2909**	**0.0357**	**0.0227**	**0.0238**	**0.0233**
	σ	2.1164	2.1589	2.1185	2.1001	2.1182
		**0.7689**	**0.0464**	**0.0245**	**0.0200**	**0.0182**
	μ	2.2546	2.1878	2.0869	2.0774	2.1753
		**1.4630**	**0.1869**	**0.0903**	**0.1211**	**0.0547**
50	θ	2.0815	2.0807	2.0739	2.0913	2.1053
		**1.3059**	**0.0135**	**0.0089**	**0.0117**	**0.0081**
	σ	2.1136	2.1078	2.1192	2.0794	2.1015
		**0.3862**	**0.0216**	**0.0193**	**0.0081**	**0.0125**
	μ	2.2288	2.0915	2.0217	2.0483	2.1582
		**0.9203**	**0.0786**	**0.0378**	**0.0982**	**0.0335**
100	θ	2.0486	2.0032	2.0023	2.0106	2.0095
		**0.4421**	**0.0050**	**0.0025**	**0.0011**	**0.0001**
	σ	2.0971	2.0011	2.0005	2.0020	2.0123
		**0.3103**	**0.0067**	**0.0034**	**0.0011**	**0.0010**
	μ	2.2081	2.0386	1.9923	2.0016	2.0353
		**0.3594**	**0.0495**	**0.0249**	**0.0106**	**0.0042**

**Table 6:** MLEs, BEs and MSEs for MWED using θ = 2, σ = 2 and μ = 1.

n		MLE	SELF	PLF	QLF	ELF
20	θ	2.0822	2.1347	2.1434	2.1223	2.0792
		**2.1371**	**0.0395**	**0.0463**	**0.0252**	**0.0136**
	σ	2.1992	2.0704	2.1158	2.1255	2.1879
		**0.9408**	**0.2108**	**0.1148**	**0.0553**	**0.0567**
	μ	1.1197	1.0848	1.1198	1.0662	1.1024
		**0.7065**	**0.1214**	**0.1241**	**0.0752**	**0.0501**
50	θ	2.0808	2.0462	2.0517	2.082	2.0792
		**1.1630**	**0.0183**	**0.0126**	**0.0130**	**0.0035**
	σ	2.1976	2.0337	2.0605	2.0954	2.1173
		**0.4824**	**0.1561**	**0.0751**	**0.0291**	**0.0227**
	μ	1.1181	1.0845	1.1057	1.0308	1.0797
		**0.5847**	**0.0541**	**0.0536**	**0.0321**	**0.0197**
100	θ	2.0007	2.0103	2.0136	2.0032	2.0039
		**0.5303**	**0.0133**	**0.0067**	**0.0024**	**0.0012**
	σ	2.1649	2.0142	2.0339	1.9758	2.0914
		**0.3812**	**0.0800**	**0.0191**	**0.0113**	**0.0145**
	μ	1.0963	1.0120	1.0250	0.9733	1.0051
		**0.2036**	**0.0147**	**0.0153**	**0.0164**	**0.0090**

**Table 7:** MLEs, BEs and MSEs for MWED using θ = 0.1, σ = 0.5 and μ = 0.1.

n		MLE	SELF	PLF	QLF	ELF
20	θ	0.1136	0.1527	0.1616	0.1211	0.1166
		**0.0666**	**0.0048**	**0.0216**	**0.2220**	**0.0685**
	σ	0.5106	0.5235	0.5291	0.5109	0.5131
		**0.4391**	**0.0064**	**0.0120**	**0.0543**	**0.0039**
	μ	0.1066	0.1151	0.1244	0.1062	0.1198
		**0.7250**	**0.0022**	**0.0192**	**0.3767**	**0.1139**
50	θ	0.1114	0.1208	0.1166	0.1085	0.1116
		**0.0535**	**0.0017**	**0.0111**	**0.1947**	**0.0095**
	σ	0.5065	0.5140	0.5098	0.5095	0.5122
		**0.2225**	**0.0041**	**0.0077**	**0.0472**	**0.0011**
	μ	0.1051	0.1133	0.1172	0.1035	0.1041
		**0.4141**	**0.0015**	**0.0171**	**0.2523**	**0.0719**
100	θ	0.1096	0.1072	0.1103	0.1009	0.1048
		**0.0300**	**0.0004**	**0.0035**	**0.1149**	**0.0066**
	σ	0.4925	0.5071	0.5026	0.5005	0.5039
		**0.1522**	**0.0003**	**0.0005**	**0.0296**	**0.0005**
	μ	0.0950	0.1115	0.1130	0.1014	0.1010
		**0.2253**	**0.0006**	**0.0049**	**0.1452**	**0.0195**

**Table 8:** MLEs, BEs and MSEs for MWED using θ = 0.1, σ = 0.5 and μ = 0.5.

n		MLE	SELF	PLF	QLF	ELF
20	θ	0.1137	0.1036	0.1278	0.1080	0.1310
		**0.3843**	**0.0054**	**0.0491**	**0.1314**	**0.1320**
	σ	0.5108	0.5122	0.5134	0.5286	0.5107
		**0.3117**	**0.0081**	**0.0064**	**0.0289**	**0.0094**
	μ	0.5320	0.5238	0.5178	0.5384	0.5091
		**1.1339**	**0.0364**	**0.0369**	**0.4839**	**0.0588**
50	θ	0.1111	0.1034	0.1163	0.1034	0.1097
		**0.1663**	**0.0012**	**0.0110**	**0.1210**	**0.0732**
	σ	0.5090	0.5084	0.5108	0.5196	0.5092
		**0.2981**	**0.0026**	**0.0034**	**0.0153**	**0.0070**
	μ	0.5197	0.5136	0.5130	0.5132	0.5035
		**0.7611**	**0.0179**	**0.0211**	**0.2812**	**0.0406**
100	θ	0.1069	0.1013	0.1095	0.1021	0.1056
		**0.1137**	**0.0001**	**0.001**	**0.0483**	**0.0261**
	σ	0.5054	0.5011	0.503	0.5152	0.5003
		**0.1661**	**0.0001**	**0.0017**	**0.0069**	**0.0033**
	μ	0.4964	0.5055	0.5013	0.5058	0.5009
		**0.1150**	**0.0079**	**0.0152**	**0.2412**	**0.0298**

**Table 9:** MLEs, BEs and MSEs for MWED using θ = 0.5, σ = 0.5 and μ = 0.5.

n		MLE	SELF	PLF	QLF	ELF
20	θ	0.5181	0.5428	0.5364	0.5327	0.5293
		**3.2499**	**0.0885**	**0.1442**	**0.0820**	**0.1222**
	σ	0.5061	0.5234	0.5368	0.5334	0.5244
		**0.3035**	**0.0186**	**0.0356**	**0.0462**	**0.036**
	μ	0.5234	0.5315	0.5272	0.5146	0.5085
		**0.3734**	**0.0346**	**0.0647**	**0.1195**	**0.0705**
50	θ	0.5031	0.5400	0.5330	0.5234	0.5116
		**1.8339**	**0.0445**	**0.0203**	**0.0406**	**0.1078**
	σ	0.5051	0.5198	0.5318	0.5194	0.5199
		**0.2001**	**0.0125**	**0.0047**	**0.0253**	**0.0275**
	μ	0.5141	0.5259	0.5228	0.5112	0.5031
		**0.2786**	**0.0216**	**0.0190**	**0.0869**	**0.0457**
100	θ	0.4988	0.5051	0.5058	0.5145	0.5006
		**0.3804**	**0.0138**	**0.0120**	**0.0208**	**0.0939**
	σ	0.4907	0.5088	0.5118	0.5059	0.5099
		**0.1052**	**0.0039**	**0.0026**	**0.0142**	**0.0103**
	μ	0.4996	0.5065	0.5130	0.5031	0.4998
		**0.1122**	**0.0078**	**0.0114**	**0.0257**	**0.0080**

**Table 10:** MLEs, BEs and MSEs for MWED using θ = 0.5, σ = 0.5 and μ = 0.1.

n		MLE	SELF	PLF	QLF	ELF
20	θ	0.5068	0.5137	0.5465	0.5464	0.5366
		**0.6290**	**0.0988**	**0.0311**	**0.2118**	**0.0738**
	σ	0.5259	0.5165	0.5254	0.5116	0.5131
		**0.3110**	**0.0318**	**0.0443**	**0.0244**	**0.0370**
	μ	0.1147	0.1272	0.1298	0.1123	0.1215
		**0.2327**	**0.0041**	**0.0212**	**0.3981**	**0.1049**
50	θ	0.5027	0.5119	0.5228	0.5320	0.5347
		**0.5373**	**0.0802**	**0.0291**	**0.1069**	**0.0246**
	σ	0.5114	0.5128	0.5210	0.5083	0.5099
		**0.2980**	**0.0312**	**0.0376**	**0.0105**	**0.0353**
	μ	0.1130	0.1233	0.1164	0.1117	0.1206
		**0.1203**	**0.0028**	**0.0153**	**0.3981**	**0.0616**
100	θ	0.4969	0.5065	0.5013	0.5029	0.5094
		**0.1887**	**0.0098**	**0.0013**	**0.0288**	**0.0200**
	σ	0.5065	0.5052	0.5163	0.5009	0.5007
		**0.2128**	**0.0156**	**0.0076**	**0.0082**	**0.0220**
	μ	0.1043	0.1059	0.1019	0.1032	0.1060
		**0.1023**	**0.0002**	**0.0003**	**0.1820**	**0.0128**

**Table 11:** MLEs, BEs and MSEs for MWED using θ = 0.5, σ = 0.1, and μ = 0.1.

n		MLE	SELF	PLF	QLF	ELF
20	θ	0.5142	0.5315	0.5202	0.5435	0.5405
		**7.7511**	**0.0900**	**0.0788**	**0.3361**	**0.1927**
	σ	0.1152	0.1260	0.1320	0.1211	0.1143
		**0.0371**	**0.0028**	**0.0355**	**0.3459**	**0.0795**
	μ	0.126	0.1229	0.1252	0.1322	0.1275
		**0.4915**	**0.0043**	**0.0448**	**0.415**	**0.1644**
50	θ	0.5082	0.5288	0.5171	0.5250	0.5311
		**4.4693**	**0.0427**	**0.0353**	**0.2698**	**0.1711**
	σ	0.1046	0.1194	0.1221	0.1116	0.1096
		**0.031**	**0.0018**	**0.0123**	**0.1660**	**0.0525**
	μ	0.1233	0.1173	0.1202	0.1117	0.1187
		**0.1764**	**0.0024**	**0.0324**	**0.1825**	**0.1461**
100	θ	0.4991	0.5195	0.5035	0.5139	0.5118
		**3.217**	**0.0163**	**0.0300**	**0.1976**	**0.1015**
	σ	0.1006	0.1115	0.1156	0.1012	0.1003
		**0.0128**	**0.0012**	**0.0095**	**0.0803**	**0.0131**
	μ	0.1053	0.1002	0.1092	0.0937	0.1142
		**0.1354**	**0.0005**	**0.0058**	**0.0876**	**0.0731**

**Table 12:** MLEs, BEs and MSEs for MWED using θ = 0.1, σ = 0.1 and μ = 0.1.

n		MLE	SELF	PLF	QLF	ELF
20	θ	0.1132	0.1319	0.1176	0.1350	0.1351
		**0.5643**	**0.0183**	**0.0934**	**0.1644**	**0.1861**
	σ	0.1151	0.1362	0.1464	0.0828	0.112
		**0.0624**	**0.0066**	**0.0431**	**0.1997**	**0.4868**
	μ	0.1266	0.1313	0.1344	0.1146	0.1105
		**0.6851**	**0.0065**	**0.0458**	**0.0839**	**0.5767**
50	θ	0.1082	0.1289	0.1086	0.1046	0.1314
		**0.3488**	**0.0056**	**0.0345**	**0.1151**	**0.1323**
	σ	0.1107	0.1158	0.1274	0.0651	0.1109
		**0.0467**	**0.0031**	**0.0240**	**0.1593**	**0.2792**
	μ	0.1165	0.1175	0.1343	0.1136	0.1103
		**0.4343**	**0.0045**	**0.0349**	**0.0651**	**0.5162**
100	θ	0.1043	0.1199	0.1009	0.0681	0.1012
		**0.1931**	**0.0027**	**0.0166**	**0.0516**	**0.0929**
	σ	0.1025	0.1094	0.1196	0.0580	0.1041
		**0.0241**	**0.0019**	**0.0116**	**0.1321**	**0.1061**
	μ	0.1051	0.1109	0.1174	0.0736	0.1036
		**0.1380**	**0.0013**	**0.0211**	**0.0159**	**0.2265**

The steps to generate the right censored simulated samples and to compute estimates have been given in the following.

*Step 1*: Generate a sample of size ‘n’ from MWED using inverse transformation technique.

*Step 2*: Sort the generated sample in ascending order of magnitudes of the values.

*Step 3*: Decide the censoring rate, that is, what number/proportion of values will be censored.

*Step 4*: Let we have starting ‘r’ number of items are completely observed, then remaining ‘n –r’ number of items are assumed censored.

*Step 5*: Take rth observed item as the value of x_r_.

*Step 6*: Apply the LA given in Section “Lindley’s Approximation (LA)” to obtain the numerical estimates.

*Step 7*: Repeat Step-1 to Step-6 10,000 times and report the average of the estimates and their MSEs.

**Figure 1:**
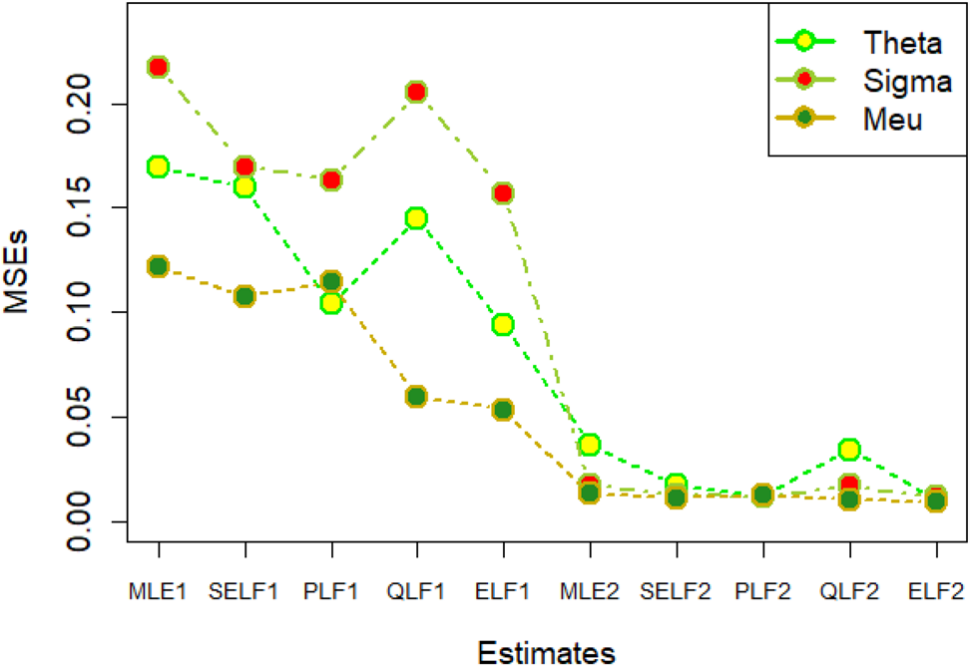
MSEs for estimates using θ = 1, σ = 1, μ = 1.

**Figure 2:**
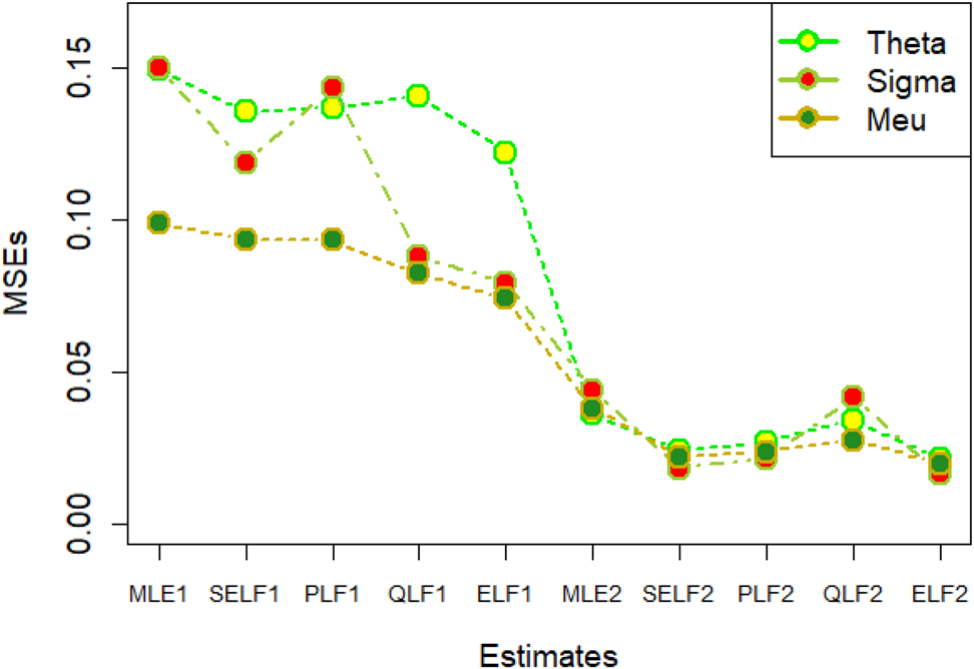
MSEs for estimates using θ = 1, σ = 2, μ = 1.

**Figure 3:**
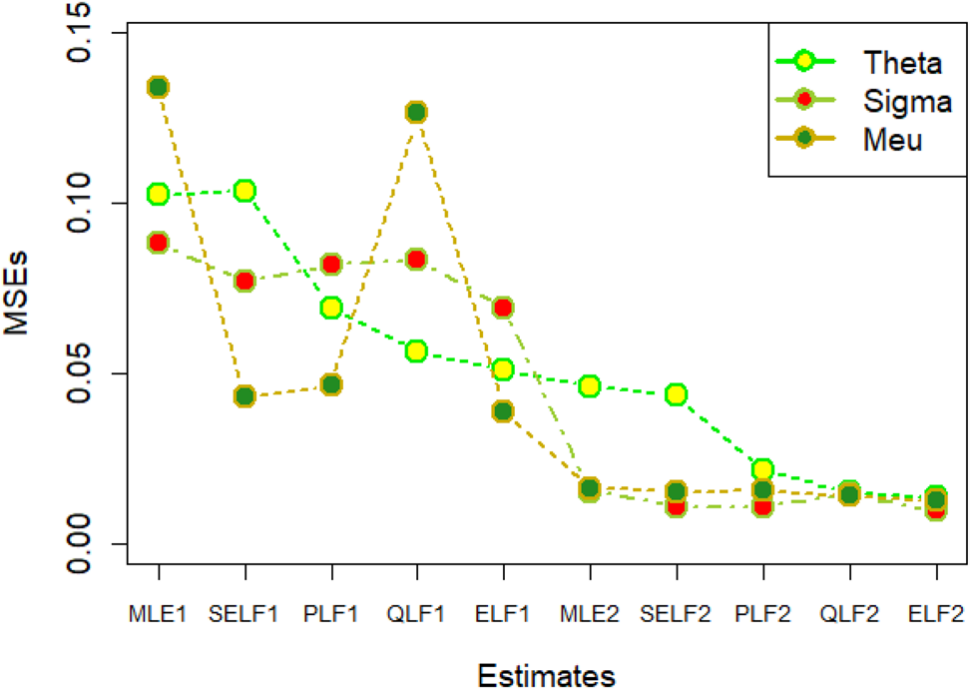
MSEs for estimates using θ = 1, σ = 2, μ = 2.

**Figure 4:**
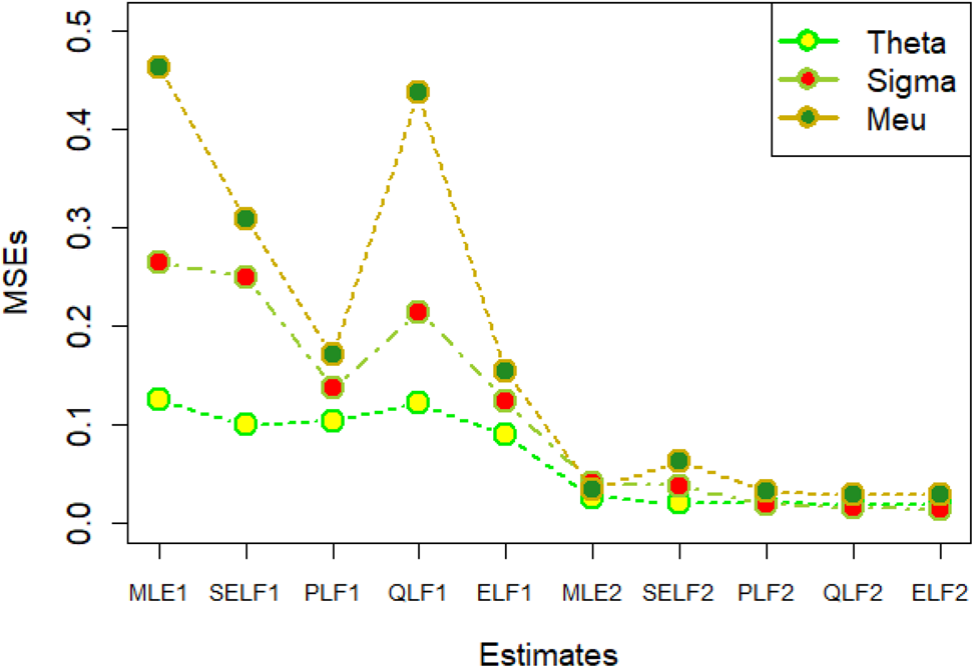
MSEs for estimates using θ = 1, σ = 2, μ = 2.

**Figure 5:**
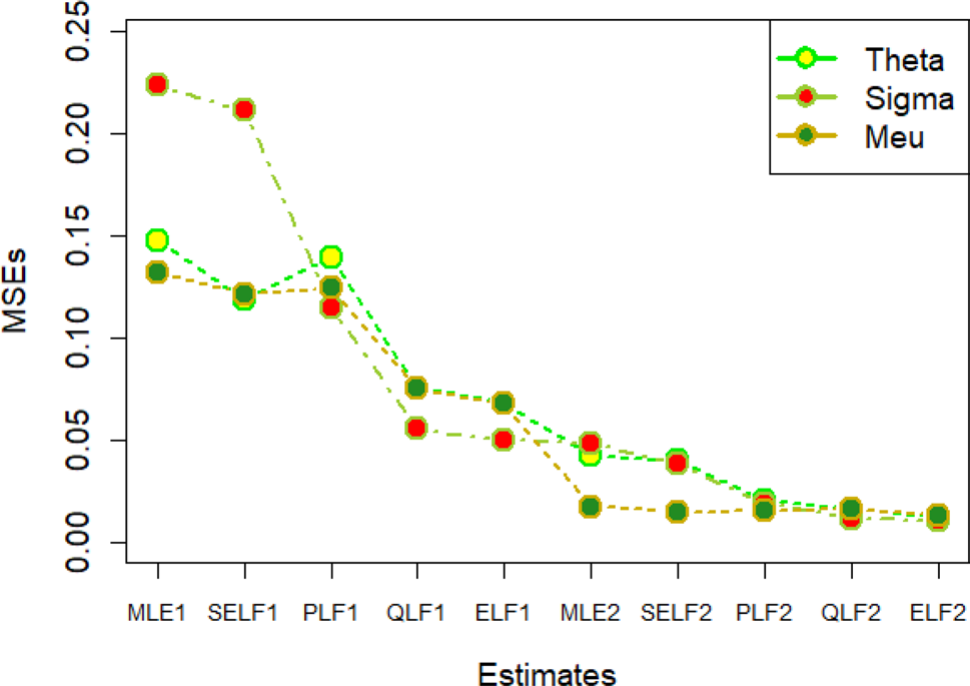
MSEs for estimates using θ = 2, σ = 2, μ = 1.

**Figure 6:**
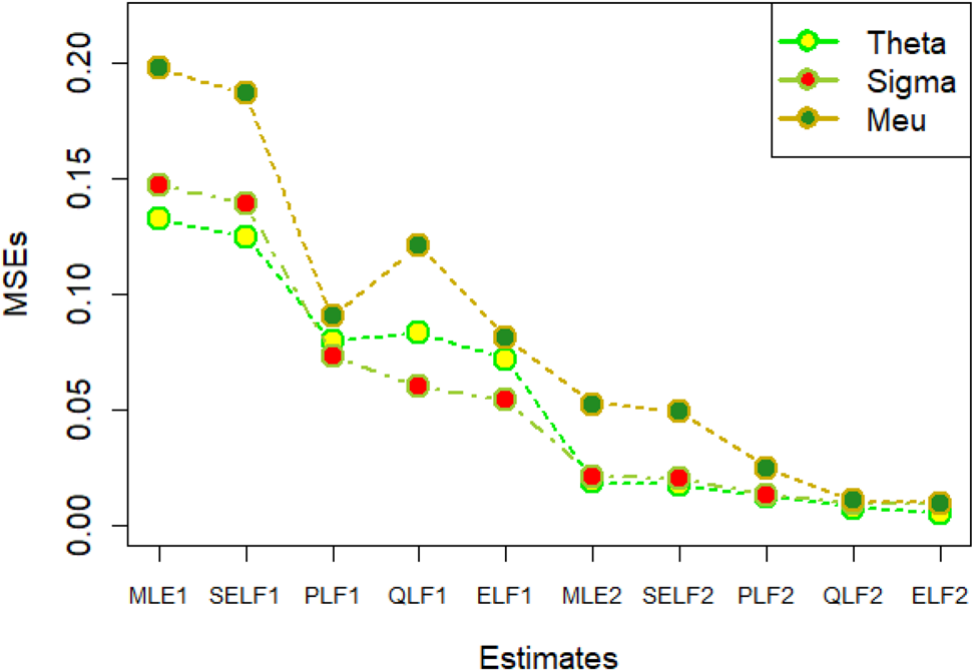
MSEs for estimates using θ = 2, σ = 2, μ = 2.

**Figure 7:**
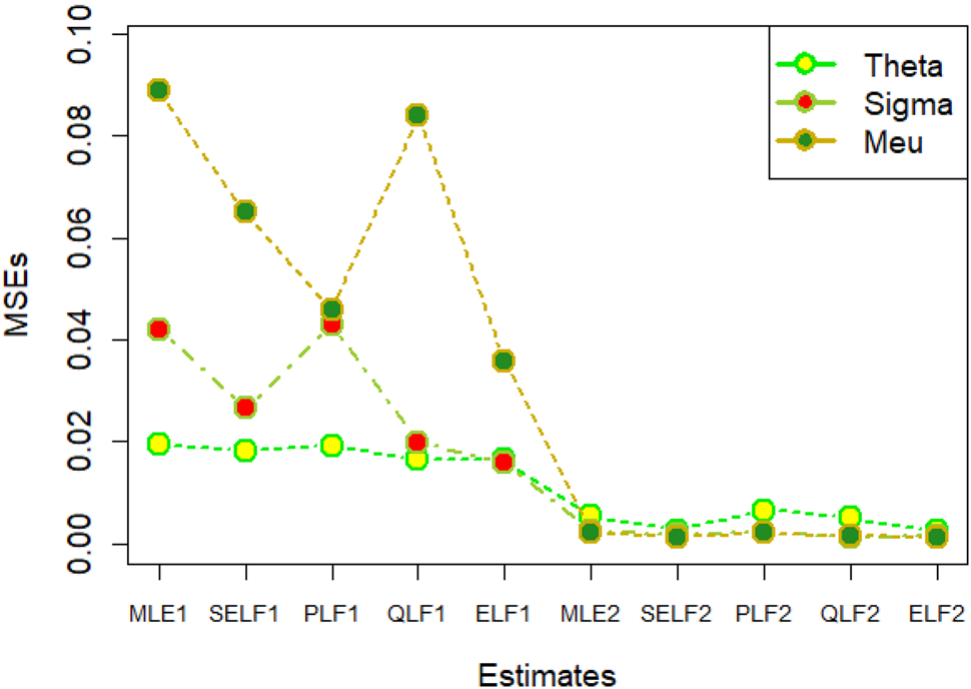
MSEs for estimates using θ = .1, σ = .1, μ = .1

**Figure 8:**
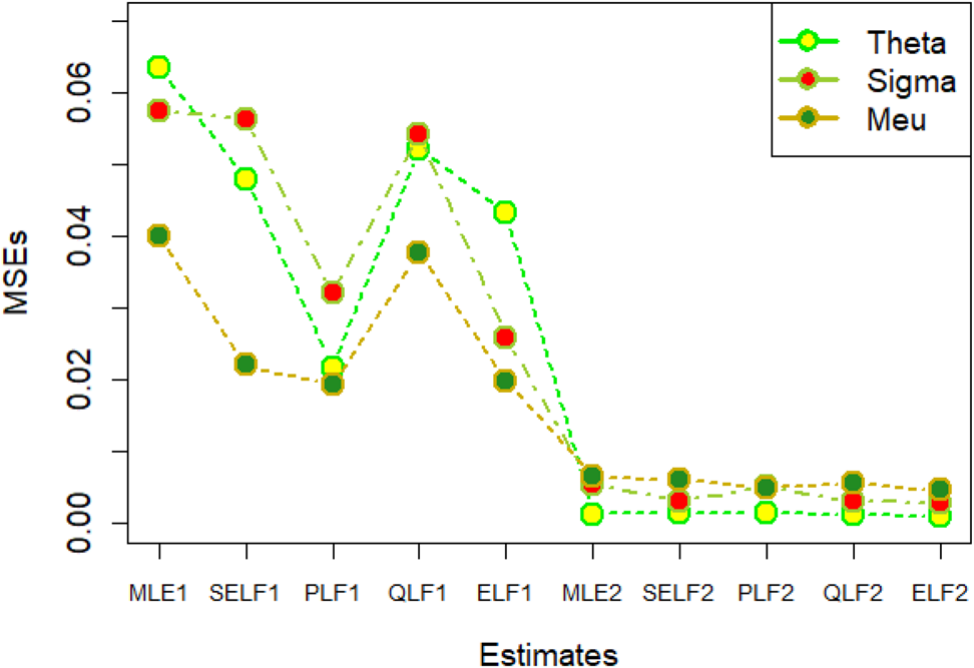
MSEs for estimates using θ = .1, σ = .5, μ = .1

**Figure 9:**
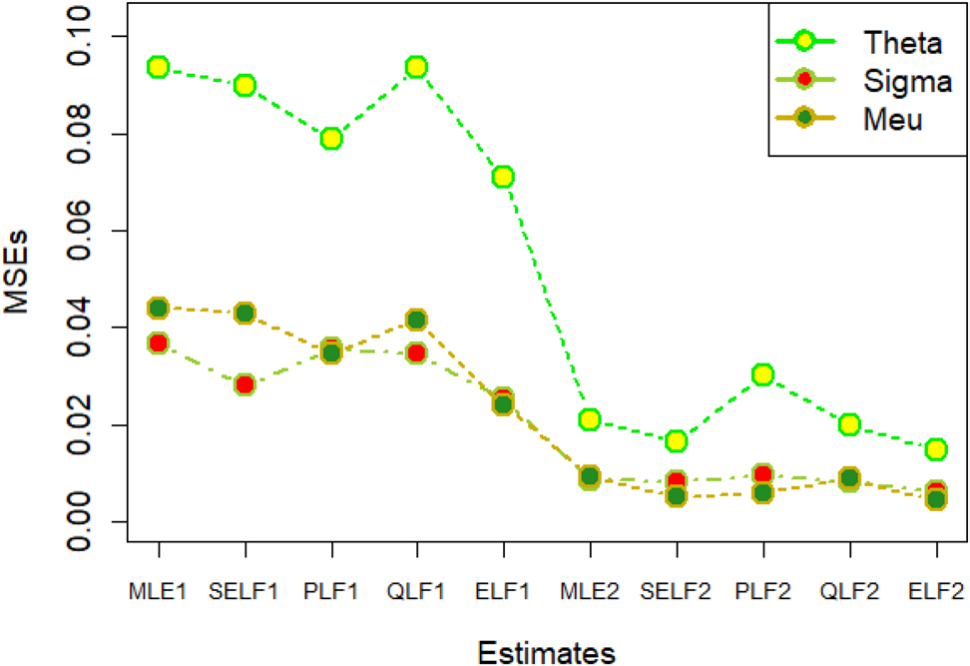
MSEs for estimates using θ = .5, σ = .1, μ = .1

**Figure 10:**
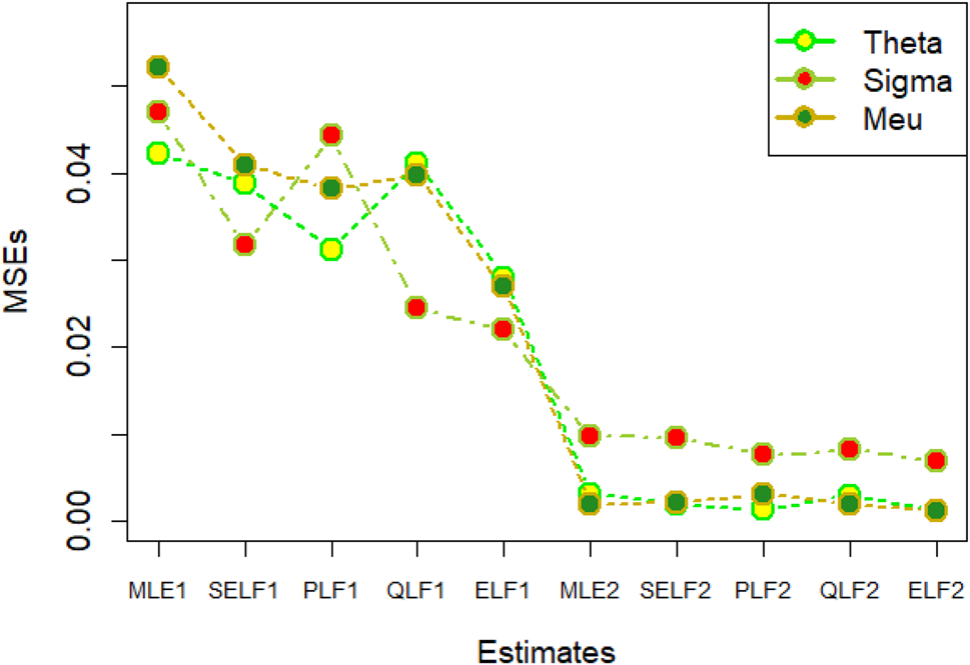
MSEs for estimates using θ = .5, σ = .5, μ = .1

**Figure 11:**
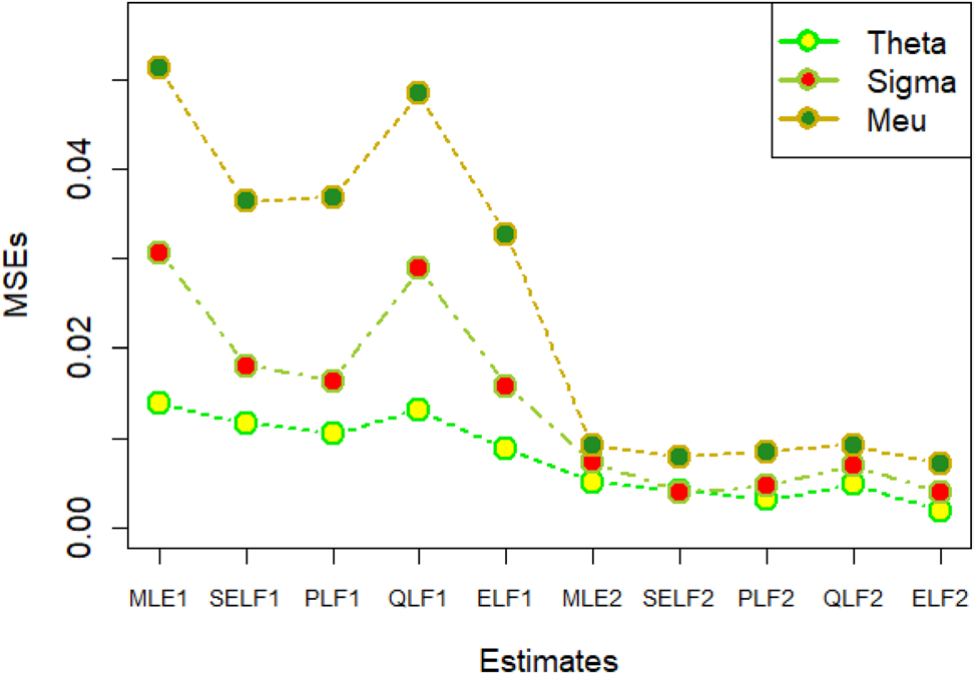
MSEs for estimates using θ = .1, σ = .5, μ = .5

**Figure 12:**
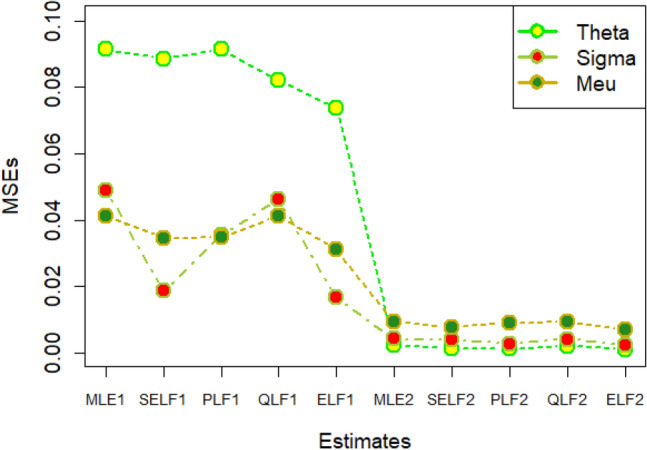
MSEs for estimates using θ = .5, σ = .5, μ = .5

The graphs for amounts of MSEs associated with estimates using simulated datasets of size n = 20 and 100 for different parametric values, have been placed in Figs. 1, 2, 3, 4, 5, 6, 7, 8, 9, 10, 11 and 12. In the said figures, MLE1 along X-axis represents the amount of MSEs associated with estimates using n = 20, while MLE2 indicates the amount of MSEs under SELF, PLF, QLF and ELF using n = 20 have represented by SELF1, PLF1, QLF1 and ELF1. On the other hand, the amounts of MSEs under different loss functions, for n = 100 have been denoted by SELF2, PLF2, QLF2 and ELF2. From Figs. 1, 2, 3, 4, 5, 6, 7, 8, 9, 10, 11 and 12, it can be assessed that the amounts of MSEs for n = 100 are considerably smaller as compared to those for n = 20. Hence, the MSEs tend to decrease by increasing the sample size. Further, the amounts of MSEs under Bayesian estimation are smaller than those under MLEs, in majority of the cases, especially in the small samples (n = 20). Additionally, the amounts of MSEs are the minimum under ELF, with only few exceptions. Hence, the results from simulate censored datasets revealed that the posterior estimation under ELF can provide gains in efficiencies for estimating the model parameter from MWED, especially in the small samples.

### Applicability of MWED in modeling censored medical datasets

This section explores the applicability and suitability of MWED to model censored medical datasets. Two real datasets have been utilized for this purpose. The first dataset is about remission times (in weeks) of 30 leukemia patients having a particular type of therapy. The observations of the said dataset are: 1, 1, 2, 4, 4, 6, 6, 6, 7, 8, 9, 9, 10, 12, 13, 14, 18, 19, 24, 26, 29, 31*, 42, 45*, 50*, 57, 60, 71*, 85* and 91. The starred values (*) represent the censored times. This dataset has been named as D1. The second dataset is about the survival times (in days) of 22 bile duct cancer patients having radiation and drug treatment. The survival times are as follows: 30, 67, 79*, 82*, 95, 148, 170, 171, 176, 193, 200, 221, 243, 261, 262, 263, 399, 414, 446, 446*, 464 and 777. These data has been named as D2. Both of the datasets have been reported by Lawless^25^.

**Figure 13:**
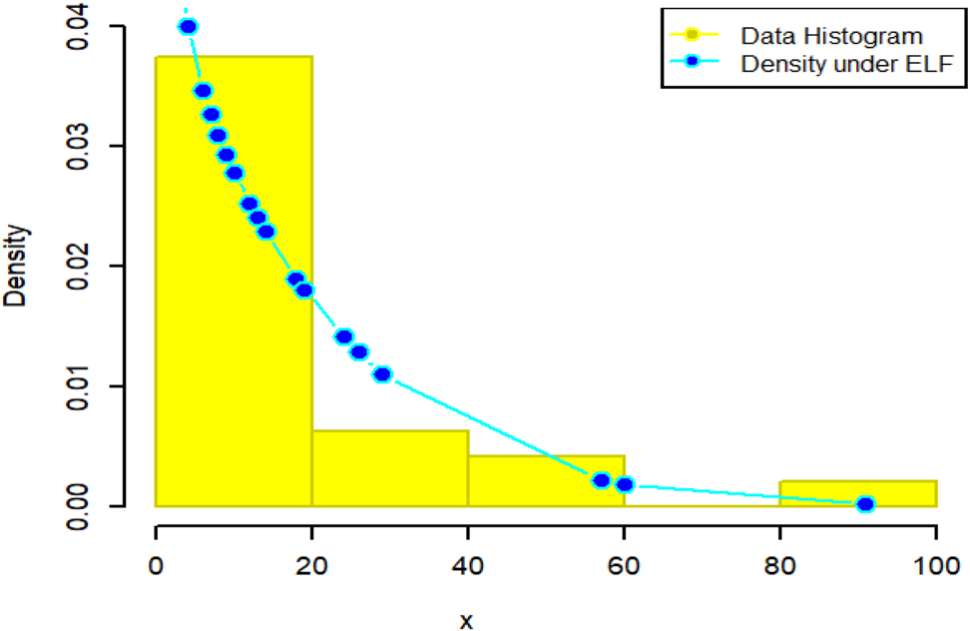
Density curve for censored real D1.

**Figure 14:**
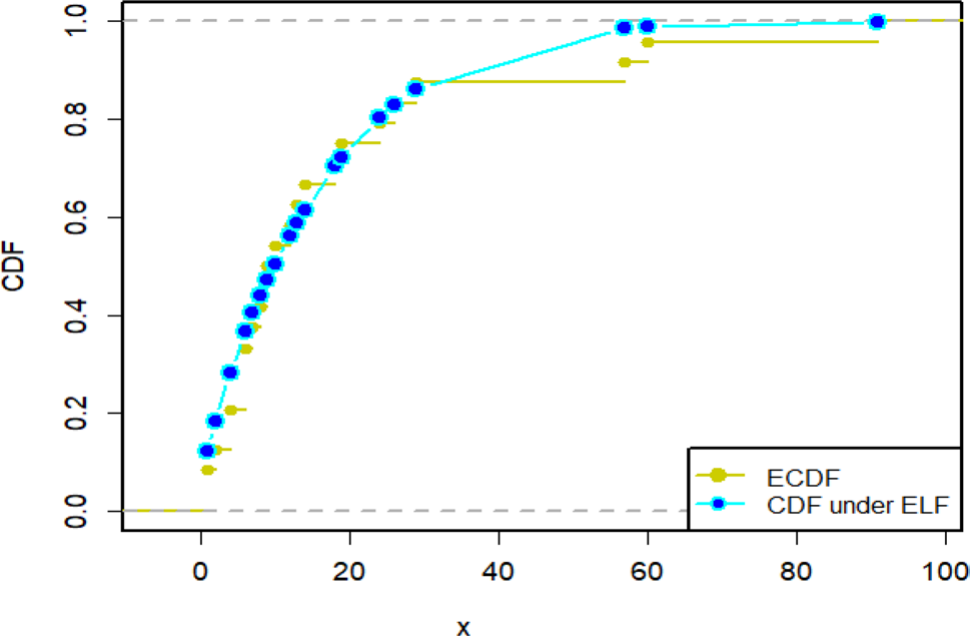
CDF plot for censored real D1.

**Figure 15:**
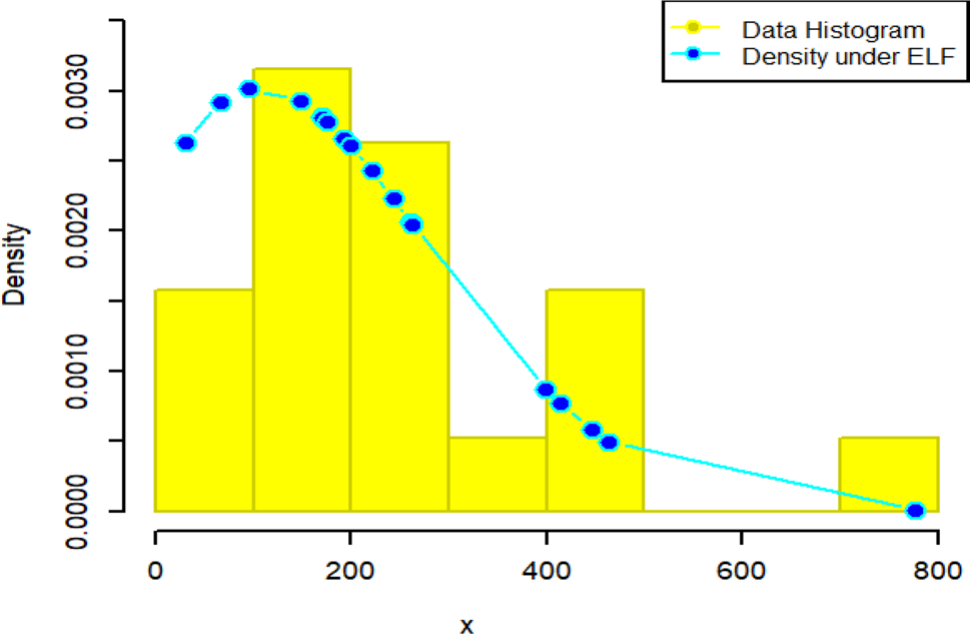
Density curve for censored real D2.

**Figure 16:**
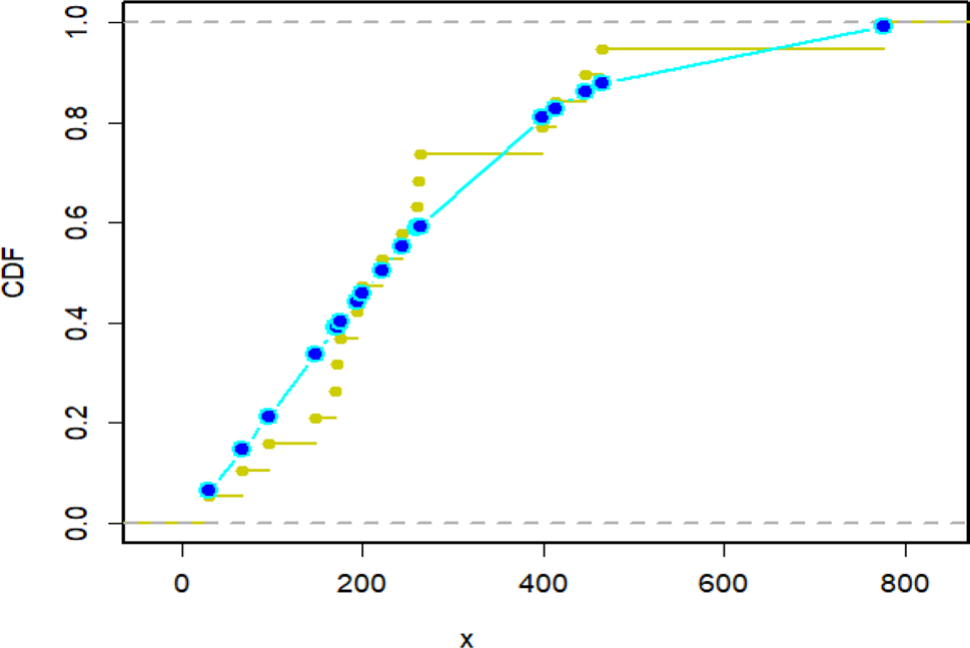
CDF plot for censored real D2.

Since the estimation under ELF outperformed its counterparts in the simulation study, we have reported the description of estimates under ELF in detail. For that purpose, the density plots and CDF plots for two censored real medical datasets have been reported in Figs. 13, 14, 15 and 16. These Figures indicate that the estimates under ELF have been quite efficient in describing the behavior of each real datasets. This is due to the fact that estimated density curves and CDF curves, under ELF, are quite closer to the corresponding empirical curves.

**Figure 17:**
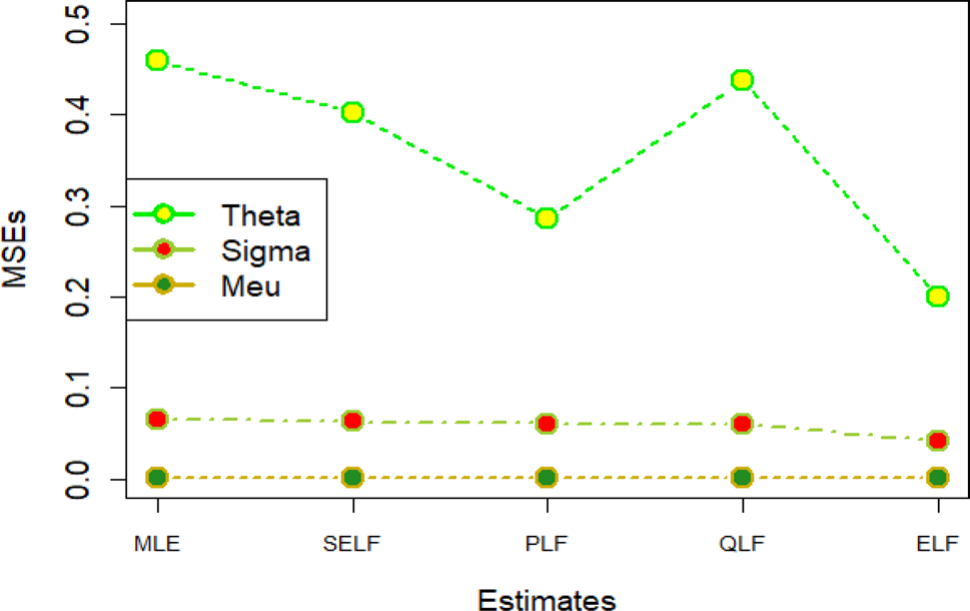
MSEs for estimates using D1.

**Figure 18:**
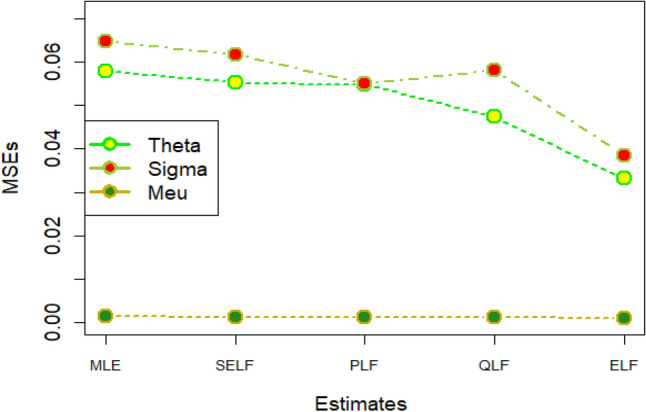
MSEs for estimates D2.

**Figure 19:**
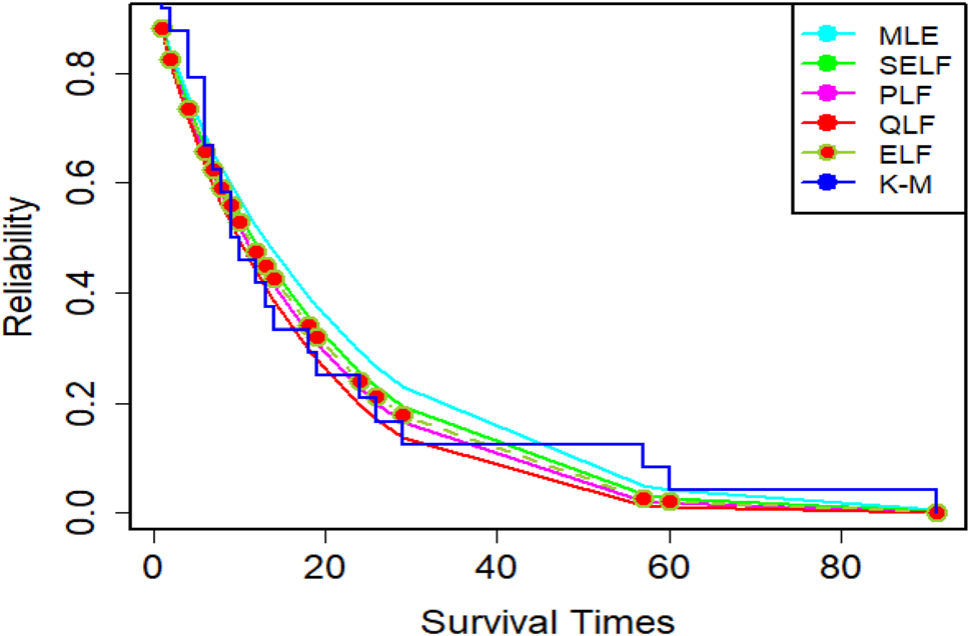
Reliability plot for censored real D1.

**Figure 20:**
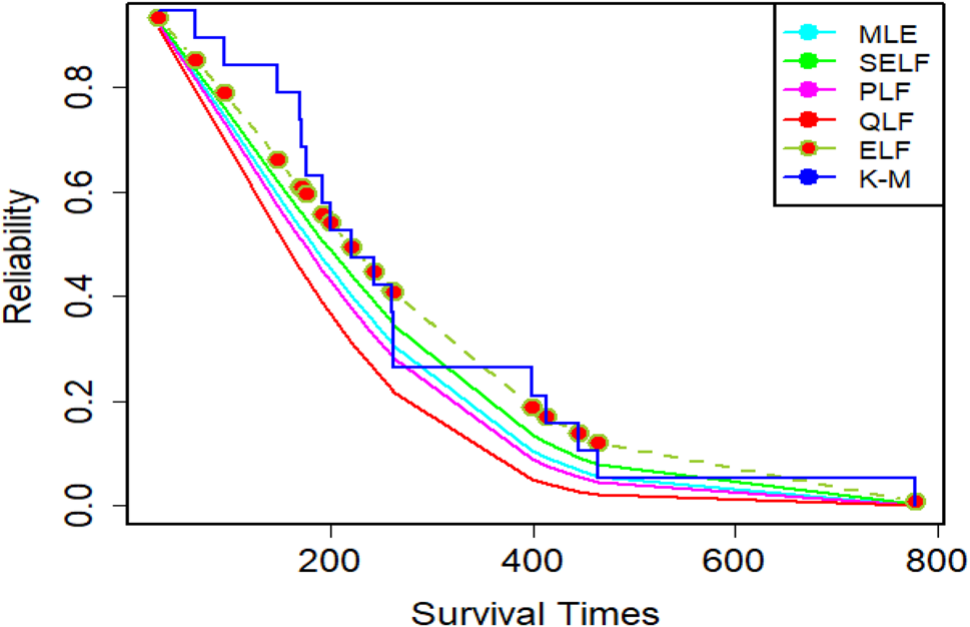
Reliability plot for censored real D2.

The amounts of MSEs associated with estimates under MLE and Bayesian methods, using two censored real medical datasets, have been presented in Figs. 17 and 18, respectively. These Figures elucidate that all the estimation methods have provided satisfactory estimates. However, the estimates under ELF are slightly better than those under MLE, SELF, QLF and PLF. On the other hand, the reliability functions for MWED using both datasets have been given in Figs. 19 and 20, respectively. From these figures, it can be assessed that the survivors of the patients are more accurately modeled using the Bayes estimates under ELF. The efficiency of the proposed estimators in modeling the survivors of the patients is more evident in Fig. 19 developed for 30 leukemia patients.

## Discussions

The MWED is very important distribution to model failure times and reliability of the data. It is often preferred over other modifications of the Weibull distribution owing to the fact that the model possesses closed form CDF and hazard rate. The use of MWED is especially advantageous when the hazard rate of the data is of bathtub shape. The models with closed form CDF and hazard rate are also preferred to model the censored datasets. It is worth mentioning here that the survival times of the patients often possess bathtub shaped hazard rate (Kayid^26^). So, the MWED having bathtub shaped hazard rate is very relevant in modeling the survival times and reliability of the patients. However, according to the best of our knowledge, no earlier study has reported this aspect of MWED. In addition, the Bayesian analysis of the censored datasets using different modifications of the Weibull distribution has been quite frequent in literature. A careful review of the literature suggests that the Bayesian analysis of censored datasets using MWED has not been discussed in detail in literature. Especially, the suitability of the MWED in modeling censored medical datasets using Bayesian methods has not been discussed in literature. The gap has been bridged, in this paper, by considering Bayesian analysis of the censored medical datasets using MWED. The detailed simulation study suggests that the estimates based on MWED possess the consistency property. The estimates using Bayesian methods were found to be better than those under MLE method. In case of Bayesian methods, the estimates under ELF were quite better as compared to their counterparts. These finding are in agreement with the earlier studies conducted for generalized exponential distribution (Mitra abd Kundu^27^) and for Weibull model (Kundu^28^). The suitability of the MWED in modeling censored medical datasets was evaluated by modeling two right censored datasets regarding survival times of the cancer patients. It was encouraging to observe that MWED was able to represent the behavior of both the datasets. Hence, MWED is a very suitable candidate model to analyze the censored medical datasets. The efficiency of the estimates based on MWED can further be improved by employing Bayesian methods in place of MLE method.

## Conclusion

Although the literature contains the analysis of censored medical datasets using the modified versions of the lifetime distributions, most of the proposed models were not modifications of the Weibull distribution. Especially the Bayesian estimation of censored medical datasets using the modified version of Weibull distribution is rarely found in literature. The Weibull distribution is very important lifetime model and many authors have proposed different modifications of this model. The recent modification of Weibull distribution, namely MWED, has been shown to perform better than Weibull and mixture of Weibull distribution in modeling lifetime datasets. We have proposed Bayesian analysis of censored medical datasets using MWED. The results have been compared with most frequently used MLE method. The informative priors and different loss functions have been used for the analysis. The reliability characteristics of the said datasets have also been evaluated. The detailed simulation study has been conducted to prove the consistency and efficiency of the proposed estimates as compared to MLE. The applicability and suitability of the MWED is modeling censored medical datasets has been explored using two real datasets.

The results confirmed the consistency property of the estimates. In addition, the performance of the Bayes estimates was better as compared to MLE. This feature of Bayes estimates was more evident in the small samples. In particular, the Bayes estimates under ELF and informative prior were the best. The proposed estimators were quite insensitive with respect the different choices of true parametric values. Further, the performance of the proposed Bayes estimates, in modeling the censored real medical datasets, was better as compared to their counterparts. In particular, the survivors of the patients are more accurately modeled using the Bayes estimates under ELF. Finally, the MWED was explored to be a very potential candidate for modeling censored medical datasets. The proposed model was able to represent the behavior of both censored real medical datasets. The study is useful for the researchers dealing with censored medical datasets, especially when more flexibility in modeling is needed.

## Supplementary Information

Supplementary Information.

## Data Availability

All data generated or analyzed during this study are included in this published article.
