# Author Correction: Applicability of modified weibull extension distribution in modeling censored medical datasets: a bayesian perspective

**DOI:** 10.1038/s41598-022-25158-6

**Published:** 2022-11-29

**Authors:** Navid Feroze, Uroosa Tahir, Muhammad Noor-ul-Amin, Kottakkaran Sooppy Nisar, Mohammed S. Alqahtani, Mohamed Abbas, Rashid Ali, Anuwat Jirawattanapanit

**Affiliations:** 1 Department of Statistics, The University of Azad Jammu and Kashmir, Muzaffarabad, Pakistan; 2 Department of Statistics, COMSATS University Islamabad-Lahore Campus, Lahore, Pakistan; 3 Department of Mathematics, College of Arts and Sciences, Prince Sattam Bin Abdulaziz University, Wadi Aldawaser, Saudi Arabia; 4 Radiological Sciences Department, College of Applied Medical Sciences, King Khalid University, Abha, 61421 Saudi Arabia; 5 BioImaging Unit, Space Research Centre, Michael Atiyah Building, University of Leicester, Leicester, LE1 7RH UK; 6 Electrical Engineering Department, College of Engineering, King Khalid University, Abha, 61421 Saudi Arabia; 7 Electronics and communications Department, College of Engineering, Delta University for Science and Technology, Gamasa, 35712 Egypt; 8 School of Mathematics and Statistics, HNP LAMA, Central South University, Changsha, 410083 Hunan People’s Republic of China; 9 Department of Mathematics, Faculty of Science, Phuket Rajabhat University (PKRU), 6 Thepkasattree Road, Raddasa, Phuket, 83000 Thailand

Correction to: *Scientific Reports* 10.1038/s41598-022-21326-w, published online 13 October 2022

In the original version of this Article Anuwat Jirawattanapanit was incorrectly affiliated with ‘Faculty of Science and Technology, Rajamangala University of Technology Suvarnabhumi, 7/1 Village No. 1, Nonthaburi 1 Road, Suan Yai Sub District, Muang District, Nonthaburi 11000, Thailand’. The correct affiliation is listed below.

Department of Mathematics, Faculty of Science, Phuket Rajabhat University (PKRU), 6 Thepkasattree Road, Raddasa, Phuket 83000, Thailand.

Furthermore, Anuwat Jirawattanapanit was omitted as a corresponding author. Correspondence and requests for materials should also be addressed to anuwat.j@pkru.ac.th.

The original Article has been corrected.

