# Supplementary material for: Applicability of modified weibull extension distribution in modeling censored medical datasets: a bayesian perspective
Source: Sci Rep. 2022 Oct 13;12:17157. doi: 10.1038/s41598-022-21326-w (PMC9558059; doi:10.1038/s41598-022-21326-w)
Supplement: Supplementary file 1 — Supplementary Information. [file 41598_2022_21326_MOESM1_ESM.pdf]

R Code for MLEs and Plots

Library

```
library('bbmle')
```

Pdf of Modified weibull extension dist

```
fx = function(x, a, b, c){a*b*(x/c)^(b-1)*exp((x/c)^b+a*c*(1-exp((x/c)^b)))}
```

Cdf of Modified weibull extension dist

```
Fx = function(x, a, b, c){1-exp(a*c*(1-exp((x/c)^b)))}
```

Reliability function of Modified weibull extension dist

```
Rt = function(x, a, b, c){exp(a*c*(1-exp((x/c)^b)))}
```

Failure rate function of Modified weibull extension dist

```
FRt = function(x, a, b, c){a*b*(x/c)^(b-1)*exp((x/c)^b)}
```

Quantile function of the Modified Weibull extension distribution

```
qfw = function(u, a, b, c){c*(log(1-(1/(a*c))*log(1-u)))^(1/b)}
set.seed(123457) # Set the seed for reproducible results
n=70
a1=0.1
b1=0.1
c1=0.1
u = runif(n, min=0, max=1)  #Uniform Distribution
x= qfw(u, a = a1, b = b1, c = c1 ) # quantile function
summary(x)
x=c()
MLE.W <- function(a, b, c){U = sum(log(fx(x, a, b, c))); return(-U)}
MLE.W
fit.mle.W <- mle2( MLE.W, start = list( a = .1, b = 0.01, c = .1 ))
summary (fit.mle.W)
```

KS Test

```
ks.test( x, "Fx", a=coef(fit.mle.W)[1], b=coef(fit.mle.W)[2], c=coef(fit.mle.W)[3])
```

Goodness of fit Statistics

```
AIC = AIC(fit.mle.W); AIC
LL = logLik(fit.mle.W); LL
BIC = -2*LL+3*log(n); BIC
```

Plot of PDF

```
Fecdf = hist(x) #Empirical histogram of data
hat = FX(a = coef(fit.mle.W)[1], b = coef(fit.mle.W)[2], c = coef(fit.mle.W)[3], x = sort(x))
fhat = fx(a = coef(fit.mle.W)[1], b = coef(fit.mle.W)[2], c = coef(fit.mle.W)[3], x = sort(x))
x = sort(x)
windows() ; par(mfrow=c(1,1))
plot(Fecdf, xlab = "", lwd = 3, do.points = FALSE, xlim = c(min(x), max(x)), col = "7", ylab = "", main="")
par(new = TRUE)
plot(x, fhat, lty= 1, type = "l", lwd = 3, xlab = expression(x), ylab = expression(f(x)), xlim = c(min(x), max(x)), ylim = c(0, 1),col = "4")
legend(median(x)/3, 1,  legend = c( expression(Ehist), expression(Epdf)), cex = 1, lty = c(1, 2), col = c(7, 4), lwd = 3)
```

##Plot of CDF

```
Fecdf = ecdf(x) # Empirical cdf of data
hat = FX(a = coef(fit.mle.W)[1], b = coef(fit.mle.W)[2], c = coef(fit.mle.W)[3], x = sort(x))
fhat = fx(a = coef(fit.mle.W)[1], b = coef(fit.mle.W)[2], c = coef(fit.mle.W)[3], x = sort(x))
x = sort(x)
windows() ; par(mfrow=c(1,1))
plot(Fecdf, verticals = TRUE, xlab = "", lwd = 3, do.points = FALSE, xlim = c(min(x), max(x)), col = "1", ylab = "", main = "")
par(new = TRUE)
plot(x, hat, lty = 2, type = "l", lwd = 3, xlab = expression(x), ylab = expression(F(x)), xlim = c(min(x), max(x)), ylim = c(0, 1), col="3")
legend(median(x)/3, 1, legend=c(expression(ECDF), expression(CDF)), cex = 1, lty = c(1, 2), col = c(1, 3), lwd = 3)
```

Plot of Reliability function

```
Refun = Rt(x, a = coef(fit.mle.W)[1], b=coef(fit.mle.W)[2], c=coef(fit.mle.W)[3])
plot(x, Refun, lty = 2, type = "l", lwd = 3, xlab = expression(x), ylab = expression(Ret(x)), xlim = c(min(x), max(x)), ylim = c(0, 1), col = "3")
legend(median(x)/3, 1,  legend = c( expression(Ret(x))), cex = 1, lty = 2, col = 3, lwd=3)
```
